# Crowdsourcing as a tool for creating effective nudges: An example for financial oversubscription

**DOI:** 10.1073/pnas.2308129120

**Published:** 2023-10-23

**Authors:** Anna Paley, Niels van de Ven

**Affiliations:** ^a^Department of Marketing, Tilburg University, 5037AB Tilburg, the Netherlands

**Keywords:** nudge, crowdsourcing, financial health, subscription services, field experiment

## Abstract

Creating effective nudges, or interventions that encourage people to make choices that increase their welfare, is difficult to execute well. Recent work on megastudies, massive field experiments that test many interventions simultaneously, reveals that nudge effectiveness both varies widely and is difficult for experts to predict. We propose an Iterative Crowdsourcing Procedure, which uses insights from members of the target population to generate and preselect nudges prior to testing them in a field experiment. This technique can supplement existing methods or stand alone as a way to generate conditions for testing in a high-quality field experiment. We test the effectiveness of this method in addressing a challenge to effective financial management: consumer oversubscription. We first document that people have more subscriptions than they think they have and that enhancing subscription awareness makes people want to cancel some subscriptions. We then use our crowdsourcing procedure to motivate people toward subscription awareness in a field experiment (N = 4,412,113) with a large bank. We find that the crowdsourced nudges outperform those generated by the bank, demonstrating that the Iterative Crowdsourcing Procedure is a useful way to generate effective nudges.

Nudges, behavioral interventions that change the way information or choices are presented without limiting freedom of choice, are important tools that can guide people toward behavior that improves their welfare (1). However, recent research finds that nudging is difficult to do well. A meta-analysis on nudges shows that the effectiveness of nudges varies widely (2, 3). Research on megastudies, massive field experiments that test multiple interventions simultaneously, confirms this large heterogeneity suggesting that it is difficult to identify effective nudges (4, 5).

We argue that crowdsourcing, or harnessing the insights of a crowd, can help overcome a challenge in generating effective nudges. One reason underlying the heterogeneous outcomes from behavioral experiments is that experts perform poorly at predicting which nudges are most effective (5). As experts often differ from the target population (e.g., in education level), they can struggle to accurately forecast the general public’s opinions and behaviors (6). Indeed, past research has confirmed that experts and the crowd differ on what communication messages they think will be effective (7), but it is unknown whether crowdsourced nudges can outperform expert-generated nudges.

Although crowdsourcing can be a cost-effective way to quickly generate many new ideas for products or promotional messages for marketing and health communication (8, 9), the literature lacks guidelines about how to identify the best ideas from a broad range of crowdsourced options potentially leading to biased selection strategies (10). We therefore propose and empirically validate a framework for implementing crowdsourcing to create and test high-quality behavioral interventions. The Iterative Crowdsourcing Procedure (see Fig. 1) relies on members of the target population at three stages: 1) generating the initial set of ideas, 2) preselecting a promising subset of ideas, and 3) evaluating which ideas are most effective in a field experiment prior to implementation.

**Fig. 1.**
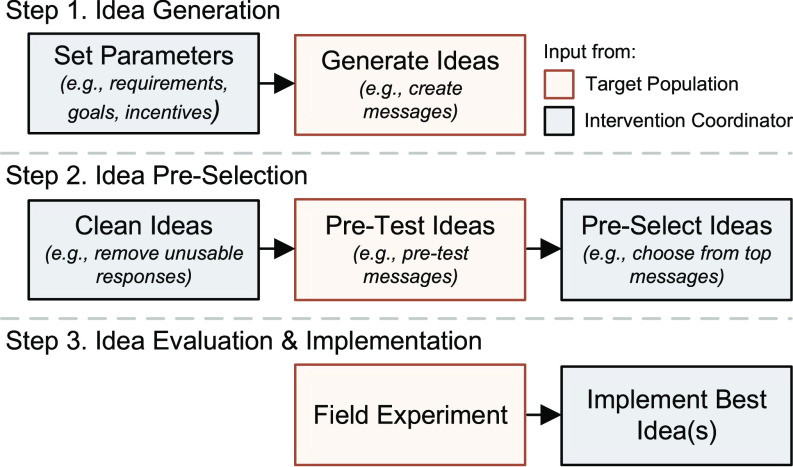
Iterative Crowdsourcing Procedure to generate, pre-select, and evaluate/implement effective nudges.

While field experimentation (step 3) is a cornerstone of behavioral research and remains a key part of our procedure, our method adds a direct way to harness insights of the crowd to create and preselect interventions (steps 1 and 2). Our procedure aims to circumvent some of the difficulty that experts face when predicting which nudges will work (5, 6). Broadly seen, existing approaches to generating interventions typically rely on some combination of expertise and exploratory research (e.g., focus groups, surveys; 11). While less theoretically grounded than these traditional approaches, the Iterative Crowdsourcing Procedure can be simpler and cheaper than doing comprehensive exploratory research (though it is likely more complex than relying on expertise alone when creating interventions). It involves less costs than other approaches for generating interventions (though there are still some costs, including those of field experimentation; see *SI Appendix* for pros and cons of the procedure). This procedure may be particularly useful for those interested in conducting behavioral experiments but lack the capacity to create high-quality interventions by identifying consumer insights, turning them into usable interventions, and selecting the most promising options (e.g., individuals and small teams, those working on tight timelines or budgets).

We employ the Iterative Crowdsourcing Procedure to help increase people’s awareness of their subscriptions, an important prerequisite of managing their financial health. Subscriptions are defined as an agreement between consumers and providers to supply a product/service continuously or at regular intervals in return for compensation. The rapid proliferation of subscriptions, growing by over 20% from 2021 to 2022 alone (12), may present concerns for consumer welfare. Specifically, people may be oversubscribed (i.e., have more subscriptions than they want) as the automatic continuation might limit their awareness of subscriptions.

## Subscription Models: A Concern for Consumer Welfare

**Fig. 2.**
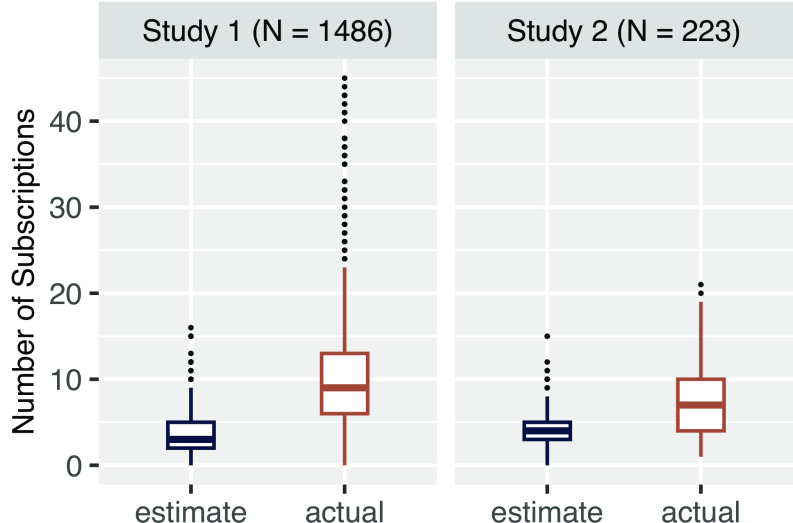
People underestimate how many subscriptions they have.

Using a Dutch nationally representative survey (from budgeting institute Nibud) and a U.S. Prolific survey, we test whether subscription awareness (awareness of one’s number of subscriptions) presents a challenge for participants (see *SI Appendix* for full method, results, and two additional studies; total N = 4,430). We use an unpacking procedure (13) that asks participants to list the subscriptions that they have within specified categories to encourage subscription awareness. We find that people initially underestimate the number of subscriptions that they have (study 1a, *M*_estimate_ = 3.75, *SD* = 2.60; *M*_actual_ = 10.47, *SD* = 6.57; *t*(1485) = 40.98, *P* < 0.001, *d* = 1.06; study 2a, *M*_estimate_ = 4.37, *SD* = 2.47; *M*_actual_ = 7.64, *SD* = 4.49; paired-*t*(222) = 15.55, *P* < 0.001, *d* = 1.04; Fig. 2) as well as their cost (*SI Appendix*). Importantly, the number of subscriptions people intend to cancel is higher after (vs. before) the subscription awareness procedure (study 2a, *M*_estimate_ = 0.97, *SD* = 1.20; *M*_actual_ = 1.48, *SD* = 1.73; paired-*t*(222) = 6.61, *P* < 0.001, *d* = 0.44).

Inspired by this challenge to financial well-being and the potential benefit of subscription awareness, we collaborated with Rabobank, a large Dutch bank, to release a feature in their mobile app that shows customers an overview of their subscriptions. This tool is an easy way to encourage subscription awareness by providing an overview of customers’ subscriptions. We preregistered the use of our Iterative Crowdsourcing Procedure for generating and preselecting message-based nudges to encourage the use of the tool. We subsequently tested the effectiveness of these crowdsourced messages against the bank’s intended message in a field experiment.

## Iterative Crowdsourcing Procedure and Results

The Iterative Crowdsourcing Procedure (Fig. 1) is guided by an intervention coordinator and follows three steps. A guide to using the procedure is available in *SI Appendix*. An intervention coordinator can be anyone with the goal of implementing nudges; whether it be an academic, marketing professional, or policy advisor.

### Step 1: Idea Generation.

First, the intervention coordinator follows the initial steps of a good behavioral insights program: they define the desired behavior and assess the feasibility of targeting the behavior (11). The intervention coordinator then sets the goals, requirements (e.g., word limit of a message), and incentive structure (e.g., bonuses for good ideas) for the crowdsourcing procedure. After this, a sample from the target population generates appropriate ideas. For our study, we recruited 200 Dutch adults and informed them about the bank’s subscription overview tool. They were asked to create a message to nudge customers toward using this new feature.

### Step 2: Idea Preselection.

This step has three phases. First, ideas that do not meet requirements are eliminated. Second, the remaining ideas are evaluated by another group of respondents from the target population on their likely effectiveness. This critical phase facilitates choosing from a large number of crowdsourced ideas by giving researchers and practitioners a way to estimate the performance of each crowdsourced option from the first step. Third, the intervention coordinator selects ideas to test in the following field test in Step 3.

For our study, 143 messages from the 200 respondents met established criteria. We also added several messages from the bank (N = 7) and ourselves (N = 4) to serve as expert-generated messages. A different sample from the target population (N = 300) evaluated 30 messages each by indicating the likelihood that they would click on the message to open the subscription overview (scale from 1 = not at all likely to 7 = extremely likely). Results revealed a wide range of clicking likelihood (M = 2.54 to 4.86). Both our top-performing message (rank 100 out of 154) and that of the bank (rank 68) underperformed relative to the top crowdsourced messages. As preregistered, we chose four of the 20 top-rated messages that differed in content to test in the field alongside the bank’s expert-generated message. These messages emphasized generating curiosity, forgotten subscriptions, possible savings, and increased control over spending (details in *SI Appendix*).

### Step 3: Idea Evaluation and Implementation.

Testing several top-ranked ideas with a field experiment is critical since the actual effectiveness of interventions may still differ from the perceived effectiveness identified in step 2, even under the best circumstances. In our case, the bank randomly distributed the four crowdsourced messages and their expert-generated message as popup ads within the bank’s app when introducing the subscription overview tool to 4,412,113 users. We tracked whether customers used the feature after seeing the message and tested whether the crowdsourced nudges persuaded customers to access their subscription overview and thereby improve their subscription awareness.

Each of the crowdsourced messages outperformed the bank’s expert-generated message (Table 1). The click-through rate for the bank’s expert-generated message (8.42%) was lower than that of the crowdsourced messages (9.99% across the four messages; χ^2^(1, N = 4,412,113) = 970.50, *P* < 0.001, OR = 1.21, 95% CI: 1.19 to 1.22). The bank’s decision to test crowdsourced messages (vs. exclusively using their own expert-generated message) increased the number of customers opening the tool by 63,585. Further, users could click a “not interested” button which prevented additional messages about the feature. The crowdsourced messages had significantly fewer users indicating that they were not interested (crowdsourced messages average: 0.52%, expert-generated message: 1.29%; χ^2^(1, N = 4,412,113) = 3,447.60, *P* < 0.001, OR = 0.40, 95% CI: 0.39 to 0.42), giving the bank an opportunity to recontact 30,772 customers compared to if they had used their own message. After the 2-wk testing phase, the bank continued with the best-performing message.

**Table 1.** Field experiment testing the effectiveness of crowdsourced messages

Message	N	CTR (OR)		Not interested (OR)	
Bank’s expert-generated message	379,489	8.42%		1.29%	
All crowdsourced versions	4,032,624	9.99%	(1.21)	0.52%	(0.44)
Crowdsourced message:					
Curiosity	1,157,502	10.57%	(1.29)	0.38%	(0.32)
Forgetting	742,726	8.68%	(1.03)	0.57%	(0.48)
Savings	1,103,064	10.19%	(1.23)	0.57%	(0.49)
Control over spending	1,029,332	10.07%	(1.22)	0.59%	(0.50)

Note: CTR = click-through rate. Odds ratio (OR) compares each crowdsourced message to the bank’s message. Each crowdsourced message differs at *P* < 0.001 compared to the bank’s message (both CTR and not interested measures).

## Discussion

This study introduces the Iterative Crowdsourcing Procedure—a methodological advancement that can help policy advisors, marketing practitioners, and academic researchers generate possible effective nudges by directly harnessing insights of the crowd. The current application of crowdsourcing focuses on persuasive messaging interventions as a nudge to improve behavior through optimizing communication. Nudges that rely on disclosing information or reminding consumers to engage in or avoid certain behaviors are ripe for crowdsourcing; however, it may be more complex to implement crowdsourcing for other types of nudges. Future research is needed to help determine for which type of nudges, in which domains, and for which target populations this crowdsourcing procedure is most effective (see *SI Appendix* for additional discussion).

Importantly, our technique can both complement existing approaches (e.g., crowdsourced interventions can be embedded in megastudies) or stand alone as a direct path to creating and testing interventions. This procedure can 1) help experts overcome potential prediction biases and 2) help make behavioral experiments more accessible by outlining a direct procedure to create possible high-quality interventions for testing. However, the method is still in its nascency, and we encourage future research to further test and refine it (see *SI Appendix* for additional discussion).

Finally, our findings document consumer oversubscription, an understated threat to financial well-being. We find that more subscription awareness increases the number of subscriptions that people would like to cancel and that our Iterative Crowdsourcing Procedure is an effective tool to increase engagement with subscription information.

## Materials and Methods

*SI Appendix* contains additional information about the Iterative Crowdsourcing Procedure as well as the methods and results of all studies. Links to preregistrations, complete methods, and data are also available in *SI Appendix*. The research was approved by the Institutional Review Board at Tilburg University; participation was voluntary, all data were collected anonymously, and informed consent was provided for the studies and waived for the field experiment.

## Supplementary Material

Appendix 01 (PDF)

## Data Availability

Anonymized data have been deposited in researchbox [#1432 (link in *SI Appendix*)]. Some data cannot be shared [Nibud data (study 1a and 1b) is proprietary].
