# Supplementary material for: Crowdsourcing as a tool for creating effective nudges: An example for financial oversubscription
Source: Proc Natl Acad Sci U S A. 2023 Oct 23;120(44):e2308129120. doi: 10.1073/pnas.2308129120 (PMC10622905; doi:10.1073/pnas.2308129120)
Supplement: Supplementary file 1 — Appendix 01 (PDF) [file pnas.2308129120.sapp.pdf]

Supporting Information for

Crowdsourcing as a Tool for Creating Effective Nudges: An Example for Financial Oversubscription.

Anna Paley* & Niels van de Ven

Corresponding Author: Anna Paley
Email: a.paley@tilburguniversity.edu

This PDF file includes:

Supporting text
SI References

Supporting Information Text

Content

1. **Guide to the Iterative Crowdsourcing Procedure** **p2**
2. **Pros and Cons of the Iterative Crowdsourcing Procedure** **p6**
3. **Subscription Studies Method Details and Results** **p8**
4. **Field Experiment Details** **p10**
5. **References** **p11**

1. Guide to the Iterative Crowdsourcing Procedure

Figure 1 from the main paper contains the steps of the Iterative Crowdsourcing Procedure we propose and test. The goal of this procedure is to generate effective interventions by using the wisdom of the crowd. Below we explain the steps and best practices at each of the 3 steps: 1) idea generation, 2) idea pre-selection, and 3) idea evaluation and implementation.

This procedure is executed by an intervention coordinator. An intervention coordinator should have the skills necessary to implement a behavioral experiment but requires no specialized skillset in executing various types of exploratory research or authoring their own interventions. For example, marketing professionals, policy advisors, consumer protection agents, behavioral scientists, and academic researchers can use this procedure to directly translate insights from the crowd into potentially effective interventions. Importantly, the crowdsourced ideas that emerge in step 2 can be tested in step 3 alongside alternative methods of intervention generation, encouraging the comparison of crowdsourced interventions against interventions generated through other means.

Step 1. Idea Generation. This step contains two phases. First, the parameters of the procedure are set by the intervention coordinator (e.g., researcher, manager, etc.). Second, the crowd generates ideas aimed to achieve the goal, consistent with the set parameters.

*Set parameters***.** The intervention coordinator identifies the desired behavior as the target, sets requirements for the intervention, and incentives for the procedure. The target should be phrased such that the outcome is observable and testable in the idea evaluation and implementation phase (Step 3). For example, the intervention coordinator may set the target to prompt customers to open a new feature within an app, to increase the number of visits to go to the gym, or to encourage medical checks. It is possible to set a more abstract target (e.g., improve attitude towards vaccines), but this is more difficult to test in the final phase (Step 3). Furthermore, given that nudges are inherently behavioral in nature, specifying a behavioral target for the nudge generation will more directly link the crowdsourced nudges to the desired outcome.

The crowdsourced ideas should be constrained so that they are feasible to implement as interventions. It is up to the coordinator to determine which dimensions of the idea should be constrained and how. For example, when soliciting text-based ideas, coordinators may choose to limit word or character count. Other text (e.g., font size, rhyming) and non-text parameters (e.g., color, which images to use) may also be specified as constraints or requirements when generating the idea, though the procedure may be most effective for simple tasks.

The intervention coordinator is encouraged to be as specific as possible when setting the requirements of the crowdsourcing task. This serves three important functions. First, it makes sure that all respondents have exactly the same information, with little room for personal interpretation of the starting point. When one is asked to "optimize a message", some people might think of an email, others think of a header, some might think of a pop-up message etc. Clearly specified parameters help create the same starting point for all idea generation. Second, it increases the likelihood that the generated ideas can ultimately be implemented. Finally, the specificity in the instructions forces the idea generators to be specific as well: An idea that is too vague (e.g., "motivate people to do X") is then clearly not an acceptable answer.

The intervention coordinator should determine an incentive structure to motivate participation and renumerate participants for their efforts, considering the expectations of the sample, as well as the length and difficulty of the task. Aside from the base rate reward for taking part, we recommend incentivizing participants with tiered bonuses based on the performance of

the ideas in the idea pre-selection phase (Step 2). As incentives, we chose tiered monetary bonuses for our test in addition to the regular payment for participation in the survey. In our study the top 2% of participants received a €10 bonus, the top 5% of participants received a €5 bonus, the top 25% of participants received a €2 bonus and the top 50% of participants received a €1 bonus.

The amount paid in bonuses should depend on the length and difficulty of the task. In our test, the median response time was under 10 minutes. Online surveys usually pay around minimum wage, so the bonuses for this survey were enough to encourage people to take the task seriously. For our case study, we recruited 200 respondents via Prolific. We paid €200 for participation, €225 in bonuses, and €70 as the fee and tax to Prolific for a total cost of around €500 to generate the ideas.

It is also possible to reward participants with non-monetary rewards, such as products or services from the company. We do not recommend this type of renumeration, since it is often less valuable for participants, and it is more difficult for the company to remain anonymous (we explain in the next phase why we think this is relevant). We recommend soliciting between 100-300 responses for idea generation. This range ensures that there will be sufficient responses after removing participants during the cleaning phase (first part of Step 2) without including too many responses for the pre-testing phase (second part of Step 2). However, this procedure can also be executed with a smaller number of responses.

Generate ideas. In this phase, ideas are solicited from members of the target population. It is optimal if participants in the crowdsourcing task are recruited from a sample that resembles the target population. This increases the likelihood that ideas generated will ultimately be effective for the target population. If the target population is difficult to access, and it is not feasible to use the target population in both Steps 1 and 2, the intervention coordinator should prioritize sampling from the target population in Step 2 (idea pre-selection). The reason for this is that good idea pre-selection in Step 2 can overcome potential bias in Step 1; while a biased idea pre-selection might undo the benefit obtained in Step 1. Intervention coordinators can collect additional information (e.g., demographic information) to assess the extent to which the sample matches the target population.

When soliciting initial ideas from the crowd, the intervention coordinator should be as transparent as possible about the task itself. Participants should be informed about the goals, parameters, and incentives decided in the previous phase by the intervention coordinator. Further, participants should be made aware of the upcoming procedure and how their idea will be used if selected. When using online platforms such as Prolific Academic or MTurk for recruiting respondents, we recommend avoiding sharing the name of the organization(s) behind the crowdsourcing task in order to prevent people's pre-existing notions of the organization(s) from limiting the creativity of the ideas generated. However, it is important to give participants a concrete explanation of the organization(s) as well as the goals of the crowdsourcing procedure so their ideas will be closely aligned with the behavioral target.

As part of this phase, intervention coordinators may choose to include some of their own ideas to test. This may be driven by curiosity, idea testing, or as validation for the crowdsourcing procedure (i.e., as control messages). If the crowdsourcing procedure is being used alongside additional exploratory techniques (e.g., focus groups, survey research, review of theoretical literature), intervention coordinators are encouraged to also include the ideas generated via these methods. In our case, we included four of our own interventions and seven from the bank.

Our own interventions were based on themes that emerged during a thorough review of prior literature and consumer surveys about people's opinions on subscriptions. These themes provided avenues for different solutions to address barriers to subscription awareness: not realizing there is a problem, lack of interest, and the potential costs and benefits of knowing. As such, our interventions were intended to boost the value of the information given. For example, to boost the subjective benefits of subscription awareness, we relied on the research showing that curiosity can encourage information-seeking, even in negative contexts (Hsee and Ruan 2016; Ruan, Hsee, and Lu 2018; Loewenstein 1994). The interventions we generated further emphasized themes of making saving money easier (Thaler, 1994), and ways to enhance curiosity by focusing on social proof (Cialdini, 2001) and realizing that one typically forgets about subscriptions to trigger more curiosity (Wojtowicz & Loewenstein, 2020).

Our interventions were as follows (Note that this text has been translated from its original language of Dutch):

1) Save on fixed costs; Do you know how many subscriptions you have? Your fixed costs are listed on this page; View your situation here
2) View your subscriptions; How many do you have? Nibud finds that 91% has more than they thought; View your situation now
3) Save on fixed costs; Nibud finds that 91% of the Dutch have more subscriptions than they thought; View your situation now
4) Overview of fixed costs; Did you forget to cancel any subscriptions? See if you can save money! View your situation now

While these four interventions do not provide a holistic view of the factors that may generate interest in subscription awareness, they are based on theoretically and empirically supported findings.

We are not aware of the procedure the bank used to create their seven messages. The interventions were created by a team of experts with a background in marketing communication and behavioral science, but it is unclear whether specific market research information was collected for the goal of executing the current study.

Step 2. Idea Pre-Selection. Step 2 is divided into three phases and involves letting people rate the generated ideas and selecting the most promising ones for further idea evaluation and implementation in Step 3.

Clean ideas. This phase involves the removal of ideas that do not meet the requirements or are not accurate. We encourage intervention coordinators to be as lenient as possible and not remove ideas simply because they do not like them. If an idea includes a spelling or grammatical error, we recommend fixing this rather than removing the idea. If ideas are similar to one another, intervention coordinators should keep both ideas since subtle differences can have large consequences in the pre-testing step (Step 2 phase 2); however, practically duplicate ideas should be limited to only one entry. In our case with 200 collected messages we had 8 messages that clearly did not meet quality standards, 5 that explicitly mentioned a company (which the bank did not want but this was not specified a priori as an exclusion criterion), and 44 that were inaccurate or misleading (promising things the new mobile feature did not deliver). In the end we retained 72% of the generated messages for further testing.

Pre-test ideas. In the pre-testing phase of this step, intervention coordinators recruit members of the population to evaluate the ideas retained from Step 1. It is preferable to recruit members of the target population as they are likely to be more accurate in predicting the behavior of the target population (Hoch 1988). First, the intervention coordinator should select a metric to use for the rating of all of the stimuli. It is recommended to rate the ideas on a single dimension or construct (rather than multiple constructs). This makes the remaining phases of the procedure more straightforward. We advise to use a measure that is as close as possible to the desired outcome of the later field experimentation. In our case, we measured people’s behavioral intention to use the new feature, as that behavior was the goal of our nudge. Specifically, we informed participants about the context, presented them with a random subset of messages, and asked “How likely is it that you would click on the link after reading this text?” on a scale from 1 = very unlikely to 7 = very likely.

The pre-testing phase must avoid being too labor intensive for participants to answer while maintaining a feasible sample size for the intervention coordinator to recruit. If a smaller number of participants are recruited, they must each evaluate a larger number of ideas. However, a very large number of participants may not be feasible to recruit. We recommend that participants see no more than 30 different ideas, but the fewer ideas evaluated the better, since in real life they see only one intervention and do not compare each intervention to other interventions as they do in this pre-testing phase. We recommend collecting at least 50 responses per idea. With these numbers (max 30 ideas rated per person, and 50 raters per idea) one needs a minimum of 167 participants for each 100 ideas tested. In our specific example, we had 300 respondents rate 30 ideas each. This took them on average 6.5 minutes. Our total cost for this step, using Prolific, was €365. We acknowledge that the pre-testing of ideas in this context

may not entirely overlap with the performance of these ideas in the field since behavioral intentions and actual behavior do not correlate perfectly. However, this gives an approximate estimate of how these ideas may perform.

Pre-select ideas. In this phase intervention coordinators take the responses from the crowdsourced pre-test and decide which ideas to test in the field. They first sort the ideas based on the metric of interest. Intervention coordinators can now select some of the top ideas for further testing in the field. We suggest that intervention coordinators, a priori, decide how many ideas are contenders for the field experiment and how they will choose these ideas.

It is recommended that the intervention coordinator examines the top-rated ideas for key themes and selects several ideas from the top-rated ideas (which are unlikely to be statistically different from one another). By doing so, the intervention coordinator can choose based on (theoretical) themes that emerge in the data. That is, intervention coordinators can use the field test to examine the performance of different ideas that map onto theoretical constructs, while staying true to the specific interventions created by the crowd. This approach of selecting themes among the top-rated ideas increases the informativeness of the field experiment. In our test, we used this technique to select among the top 20 ideas. A more data-driven alternative would be to rank all ideas on the metric of interest and identify the first idea that is significantly worse than the highest-rated idea; and choose from all messages ranked above that one. Thus, the intervention coordinator selects messages from the top set that all do not differ significantly from each other on the measured criteria.

Whether or not the messages created by the intervention team were evaluated highly during the prior idea pre-testing phase, intervention coordinators are encouraged to include a self-generated message in the field study of Step 3. This serves as an expert-generated control condition (i.e., the version they would have used if they had not used the crowdsourcing procedure) in the field test in order to provide validation for the procedure. In order to execute the most informative and effective test possible, intervention coordinators are encouraged to provide the best possible idea they can then use as a benchmark for the crowdsourcing procedure.

Step 3. Idea Evaluation and Implementation.

The final step of our procedure aligns with existing approaches for behavioral research. Specifically, the intervention coordinator can use a field experiment to evaluate (i.e., test) the pre-selected ideas. Then, the intervention coordinator can select the most promising idea for implementation.

Field experiment. It is recommended to follow best practices and test the top ideas in a field experiment. Intervention coordinators can select any number of ideas to test in the field, provided the sample size is sufficient for the test. The intervention coordinator should set out a priori guidelines about the scope of the test. This can be time-based (e.g., the test will run for one week) or participant-based (e.g., the test will reach 10,000 participants). It is of course also possible to imbed crowdsourced ideas within another a larger study targeted at behavioral interventions. For example, a megastudy that contains multiple theory-based interventions (Milkman et al., 2021; Duckworth & Milkman, 2022) could add one or several crowdsourced intervention(s) to see how it performs against interventions developed using other approaches. Including a passive control group, that does not get any intervention, can further increase the informativeness of the test.

In some cases, it may not be possible to execute a field test. For example, this may happen if the execution of such experiment is very expensive or labor-intensive (e.g., creating a new product). This may also occur when there are not enough target customers to run a study that is highly powered enough to draw conclusions based on statistical significance. In these cases, it is best to use a larger sample in the idea pre-selection step and select a response from the ideas that are not statistically significantly worse than the best performing idea rather than running a field experiment. Next, the intervention coordinator can decide from the top ideas, knowing that these are all likely to perform better than the worse rated ideas.

Implement best idea(s). At the end of the field test, the intervention coordinator should conduct statistical tests to determine which idea performs the best. If one idea outperforms the others, this idea should be used going forward. If one idea does not outperform the others, the intervention coordinator can choose to continue with more than one idea or select an idea from

the top performing ideas during the field test. Our field test with the bank ran for two weeks, after which the bank implemented the best performing idea.

2. Pros and Cons of the Iterative Crowdsourcing Procedure

Comparing methods for generating interventions. The first steps of the Iterative Crowdsourcing Procedure stand alongside other exploratory research approaches (e.g., The Behavioral Insights Team, 2022), but are specifically geared towards facilitating the process of creating high-quality interventions for behavioral experiments. Current methods for developing interventions for behavioral experiments rely on two sources of information: the expertise of experiment coordinators and additional exploratory research. We contribute an additional approach to creating behavioral interventions that directly relies on insights from the crowd. By doing so, we give experts a method to address a challenge in creating effective nudges. Specifically, our approach aims to reduce the possible biases that arise when experts predict which interventions are successful. Further, it is cheaper and faster compared to large scale comprehensive exploratory research. As such, this approach may be particularly useful for individuals or small teams whose constraints (e.g., financial, temporal, skill-based) limit their capacity to develop high-quality interventions.

Some interventions are created by experts who are executing a particular behavioral experiment based on their theoretical and practical knowledge. Many different parties may be interested in executing behavioral experiments (e.g., marketing professionals, policy advisors, consumer protection agents, behavioral scientists, or academic researchers) and all of them contribute a unique set of skills and perspectives. For example, a bank interested in releasing a new feature might create interventions based on a small team (e.g., consumer insights manager, UX designer, etc.), who brainstorm ideas and select interventions based on their existing knowledge. Although relying on expertise can be a relatively cost-effective and quick way to generate interventions, the interventions that experts predict as effective may not prove to be the most effective among the target population (Milkman et al., 2022). A crowdsourced approach has the potential to improve upon or supplement the ideas of experts alone.

Individuals or teams executing behavioral experiments may also conduct exploratory research, and indeed, comprehensive exploratory research is highly valuable for creating high-quality behavioral interventions. However, conducting comprehensive exploratory research may be time-consuming, costly, or require additional expertise. For example, specialized skillsets may be required, calling for collaboration among different parties (e.g., reviewing theoretical knowledge, running focus groups, creating surveys, analyzing responses, and turning these insights into possible interventions). In some cases, the resources necessary to conduct specialized exploratory research may not be available. The Iterative Crowdsourcing Procedure aims to provide a simplified approach that can generate and test high-quality behavioral interventions that address some of the (financial, temporal, and skill-based) challenges that may arise when creating interventions.

However, the Iterative Crowdsourcing Procedure does not entirely eliminate the financial, temporal, and skill-based costs of behavioral experiments. From a purely monetary perspective, our test validating the procedure cost a total of €865 for the payment and incentives for respondents in Steps 1 and 2. It also can cost a few days of work (especially when done for the first time). This method, although requiring no highly specialized skills, the coordinator needs to have the skills and capabilities necessary to execute a behavioral experiment. Also, the coordinator must find a way to connect with the crowd. In our case, we used Prolific Academic; however, logistically sourcing the crowd's responses requires some technical knowledge.

Next, and probably most important, crowdsourcing may not reveal the customer insights that may emerge during a more comprehensive exploratory research program (e.g., understanding the underlying causes of behavior). The lack of formal hypothesis testing limits the ability for structured learning, and the behavioral conclusions drawn from crowdsourcing are likely to be narrower, less theory-based, and less generalizable. A programmatic approach based on theory allows for more abstract learning than the crowdsourcing method does.

In sum, existing approaches to behavioral interventions combine various degrees of expertise and exploratory research. Compared to relying primarily on experts, the crowdsourcing

procedure offers more opportunity to overcome biases stemming from experts being different than the crowd; however, our procedure is more time consuming and more expensive. Compared to conducting comprehensive exploratory research, however, our procedure is likely faster and less expensive; but it offers less opportunity for broad and meaningful consumer insights. Ultimately, our procedure is an additional approach to generating effective interventions and can potentially be best used as a supplement to other approaches.

Promising contexts and possible boundary conditions. Although the Iterative Crowdsourcing Procedure can be applied broadly, some contexts and target groups may be less suited for the procedure. Relatively straightforward communication-based interventions, such as in our subscription-management field study and the COVID-19 vaccination megastudy (Milkman et al., 2022), are ideal for crowdsourcing. It is likely that crowdsourcing can be particularly useful in contexts where short messages are used to disclose information, serve as a reminder, or persuade people towards behavior change.

While the crowd may be able to generate effective interventions beyond those directly studied here (e.g., the wording of a default option), future research should consider how to best apply the method and validate its performance against other approaches. For example, it is unlikely that crowdsourcing outperforms other methodologies when asking consumers for a response to a closed-ended question (e.g., the best time of day to receive a just-in-time prompt). However, a crowdsourcing procedure may be effective at generating ideas for open-ended questions that require more creativity (e.g., the best locations or activities during which to receive a particular just-in-time prompt). Other nudge types might be more complex to use crowdsourcing for, such as those that are more theory-driven or less well-known among the target group (e.g., the effect of adding a decoy option or using anchoring to influence numeric perceptions). While it is possible that the crowd may have usable insights, the crowdsourcing procedure may need further development to prove beneficial in these settings. We see the current research as a starting point, where future work can identify for what types of nudges the crowd is more or less effective when generating interventions.

For complex situations or difficult to create interventions (e.g., television ads; nutrition information labeling), using the crowd may present additional challenges. The potential crowd that has the technical capabilities to actually create interventions that align with the goal of the campaign might again be very different from the target population, reintroducing the potential biases the method is trying to limit.

Additionally, some characteristics of members of a target population might make it ill-suited to be used for designing crowdsourcing interventions. For example, populations of people such as those with a very low IQ, those low in agreeableness, or those with a high need for uniqueness may be difficult to reach or it might be difficult for them to produce an idea. Still, it might also be the case that for these atypical populations the crowdsourcing procedure could be quite effective if they can be reached. Among target populations who are more difficult to reach (e.g., billionaires, those at risk of a particular disease, etc.), crowdsourcing may be more challenging. However, in these cases, experts might be particularly different from the population, making it especially difficult to predict the population's desires and behaviors. Further, this procedure requires the crowd to introspect about their experience (a common feature of exploratory research). In cases where the crowd is not able to introspect, the procedure may have limited benefit (Nisbett & Wilson, 1977).

In sum, we suspect that the crowdsourcing procedure works best when generating simple, creative interventions, where those who are able to create the intervention are a reflection of the target population. These propositions, among others, require additional testing and validation of the Iterative Crowdsourcing Procedure. Further testing is required to clarify when and where experts struggle to generate effective interventions (vs. the crowd) and understand the strengths and possible boundary conditions of the Iterative Crowdsourcing Procedure. The current work should be seen as a proof-of-concept which requires further validation.

While the Iterative Crowdsourcing Procedure can simplify the process of generating high-quality behavioral interventions, one of its main benefits is that it can be used alongside other approaches to generating nudges. As we mention above, it is as of yet unclear under what circumstances the procedure will be more or less effective. One great way to further the knowledge on this would be to add crowdsourced nudges to future megastudy projects (Milkman

et al., 2021; Duckworth & Milkman, 2022). This would further document if and when the crowdsourced nudges perform well and would allow for further validation of the procedure.

3. Subscription Studies Method Details and Results

Preregistrations, methods, and data are available here: https://researchbox.org/1432. We preregistered study 2a, and steps 1 and 2 of the Iterative Crowdsourcing Procedure. Step 3 of this procedure was executed and controlled by the bank; thus, no preregistration was possible. The data for studies 1a and 1b was provided by Nibud and the data is proprietary. The methods and data are available for studies 2a and 2b. Studies were approved by under IRB EXE 2022-011 and EXE 2020-036 at Tilburg University.

Study 1a and 1b.

Method. We used two waves (2019, 2021) of survey data from Nibud, the Dutch National Institute for Budget Information. The original survey was in Dutch. Participants provided demographic information and were asked to estimate the number of subscriptions that they thought they had. Then, participants engaged in an unpacking procedure to generate the number of actual subscriptions. Participants saw a list of either 61 (2019) or 66 subscription categories (2021; e.g., magazines/TV guide [physical, digital], Amusement park/zoo/museum, etc.). For each subscription category they indicated whether 1) one or more adults had such a subscription, 2) 1 or more children up to 18 years of age living at home had the subscription, 3) 1 or more children living at home from the age of 18 had the subscription, 4) the household as a whole had the subscription, or 5) the household does not have the subscription. We summed the number of subscriptions identified during the unpacking procedure (i.e., the number of categories for which option 1, 2, 3 or 4 was selected). We refer to this number as the “actual” number of subscriptions and compare this to the number of subscriptions estimated prior to the unpacking procedure. We recognize that the “actual” number of subscriptions may still underestimate the true number of subscriptions for each household. For example, the data provide a binary measure of subscriptions for each category. That is, it is not possible for participants to indicate that they have multiple subscriptions within a single category. Further, it is possible that participants did not recall additional subscriptions even after seeing the category that the subscription was part of. The methodology was the same in 2019 and 2021.

Participants. In 2019, 1531 participants completed the survey, and 2021 had 1514 respondents. We first removed 7 respondents that contained clearly nonsensical answers (e.g., a negative number of estimated subscriptions, an estimated subscription total of 1000 or more). After doing so we conducted a multivariate outlier analysis across the two dependent measures and removed 45 participants from the 2019 sample and 41 participants from the 2021 sample. The final 2019 sample included 1486 participants (M_{age} = 49.00, SD = 18.05, 51% female), the final 2021 sample included 1466 participants (M_{age} = 49.97, SD = 17.13, 51% female)

Results. A paired-sample t-test reveals that participants list having more subscriptions than they initially estimate having in both 2019 ($M_{estimate}$ = 3.75, SD = 2.60; M_{actual} = 10.47, SD = 6.57; $t(1485)$ = 40.98, $p < .001$, d = 1.06) and 2021 ($M_{estimate}$ = 3.79, SD = 2.95; M_{actual} = 13.39, SD = 10.05; $t(1465)$ = 37.32, $p < .001$, d = 0.98). The number of subscriptions seems to have increased from 2019 to 2021, however, it is difficult to draw concrete inferences from this data because Nibud added more categories in 2021 which might have increased the reported number of subscriptions.

Study 2a.

Method. Study 2a followed a within-subjects design. Upon entering the survey, all participants were informed that this study would be about their subscriptions and read the following definition: “*Subscriptions refer to a payment model, where you receive a product/service while paying for it in regular payments of the same amount over time. You can receive the product/service continuously, or at regular intervals. You can have subscriptions in a broad number of categories. We are only interested in subscriptions that you pay for. This includes both subscriptions that you pay for entirely, and subscriptions that you contribute money towards (e.g., shared subscriptions).”* Participants then estimated the number and annual cost of their

subscriptions and indicated how many of these subscriptions they would like to cancel whenever it is possible.

Participants then followed an unpacking procedure to generate the number of actual subscriptions. This procedure presented 17 different categories of subscriptions (e.g., Financial Services, Computer Software, Cultural Activities, etc.). For each category, participants first indicated whether or not they pay for one or more subscriptions within that category. Participants who indicated that they did pay for subscriptions within that category listed the name and cost of the subscription, alongside the frequency with which they pay that cost (monthly/yearly). This information was used to calculate the actual number of subscriptions and the actual cost of each participant's subscriptions. Participants listed the number of subscriptions that they inputted on that page, and after doing this for all 17 categories we provided them with the total number of subscriptions that they actually had. Participants then responded to the same cancellation question as they were previously asked, after which they indicated their age, gender, and household income.

Participants. Three hundred participants were recruited through Prolific Academic and 299 participants completed the survey. Participants were removed according to the pre-registration (N = 62). There was one deviation from pre-registered exclusions: 14 participants did not indicate the frequency with which they paid a particular subscription (e.g., monthly or yearly) so the yearly subscription cost was not possible to calculate. These additional participants were therefore excluded from further analyses. Data from 223 participants were analyzed (M_{age} = 33.51, SD = 11.36; 45.7% female, 1.3% non-binary).

Results. Results reveal that participants' estimate a fewer number of subscriptions than they list ($M_{estimate}$ = 4.37, SD = 2.47; M_{actual} = 7.64, SD = 4.49; $t(222) = 15.55$, $p < .001$, d = 1.04). Participants also estimate a lower cost of these subscriptions than they actually list ($M_{estimate}$ = \$568.67, SD = 664.45; M_{actual} = \$3732.97, SD = 3800.40; $t(222) = 12.95$, $p < .001$, d = 0.87). Participants indicate a higher intent to cancel subscriptions after seeing their actual number of subscriptions ($M_{estimate}$ = 0.97, SD = 1.20; M_{actual} = 1.48, SD = 1.73; $t(222) = 6.61$, $p < .001$, d = 0.44). After seeing their actual number of subscriptions, 16% of participants want to cancel an additional subscription.

Study 2b.

Method. Study 2b aimed to replicate the results of study 2a in a between-subjects design. Upon entering the survey participants were randomly assigned to one of two between-subjects conditions (estimated subscriptions vs. actual subscriptions). In the actual subscriptions condition, participants first completed the unpacking procedure that was used in study 2a which allows us to compute the actual number and actual cost of each participants' subscriptions. Afterwards, participants responded to the cancellation intentions question from study 2a. In the estimated subscriptions condition participants followed the initial portion of the method used in study 2a: they estimated the number of subscriptions that they had, the cost of those subscriptions, and their cancellation intentions. Participants in the estimated subscriptions condition then completed a control task asking them to list purchases in 17 different categories in order to keep the conditions as parallel as possible.

Participants. Fifteen hundred participants were recruited through Prolific Academic and 1503 participants completed the survey. Participants were removed as in study 2a (N = 248). Data from 1255 participants were analyzed (M_{age} = 37.67, SD = 13.09, 40.7% female, 2.2% non-binary, 0.4% prefer not to say).

Results. Results confirm that participants in the actual condition listed more subscriptions than participants in the estimated subscriptions condition estimated that they had ($M_{estimate}$ = 3.90, SD = 2.70; M_{actual} = 8.68, SD = 4.34; $t(1253) = 23.83$, $p < .001$, d = 1.35). Participants in the actual subscriptions condition also indicate that their subscriptions cost more than estimated by participants in the estimated subscriptions condition ($M_{estimate}$ = \$318.87, SD = 437.37; M_{actual} = \$4973.87, SD = 4076.61; $t(1253) = 29.85$, $p < .001$, d = 1.70). Participants in the actual subscriptions condition intend to cancel more subscriptions compared to participants in the estimated subscriptions condition ($M_{estimate}$ = 0.92, SD = 1.27; M_{actual} = 1.18, SD = 1.70; $t(1253) = 3.14$, $p = .002$, d = 0.17).

4. Field Experiment Details.

Method. The bank executed the field experiment. They included as the control condition the version they had intended to use, if we had not run our crowdsourcing procedure. We submitted the four experimental conditions that were selected in our Iterative Crowdsourcing Procedure. The experiment ran for two weeks between April 22th 2021 and May 6th 2021. In that time period, the first time a client opened their app after the update, they received a pop-up message advertising the new subscription overview feature. The pop-up message randomly presented one of the five messages, which included four crowdsourced messages and a single message from the bank. We are unaware of the process that the bank's communication team used to generate their control condition; however, the bank informed us that, without the crowdsourcing procedure, this is the intervention they would have used. The team creating this version consists of specialists working on the app, containing people from IT, marketing & communication, and one person with a PhD in behavioral science. There was no control group that did not receive a message; at the moment a client of the bank received the subscription overview feature in the app, they also got one of the messages from our test.

The five conditions were randomly shown to customers using the bank's random assignment software. When customers saw each message, they could either click on it to examine the bank's new feature, ignore it, or click "not interested." If participants clicked "not interested," the bank would not send them follow-up messages about this feature. Over the course of the experiment, the software gradually sent more respondents to treatments that are more successful (in getting people to click on the link to the subscription overview page). For example, the bank's expert-generated idea was less effective and therefore ended up with a lower share of the respondents than the other treatments.

Below we present each message and indicate its performance, comparing it to the bank's expert generated message that read: "New in our app. All subscriptions in one easy overview. Discover it now." That message was shown to 379,489 customers, leading to 31,937 visits to the subscription overview page (8.42% click-through rate, CTR). Furthermore, 4,883 customers (1.29%) indicated they were not interested in the feature and could therefore not be send a reminder of the feature later.

The message emphasizing generating curiosity read: "What do you pay each month? Do you know what subscriptions you have each month? See them here!" This message was sent to 1,157,502 customers and generated 122,394 clicks (CTR = 10.57%), which is better than the expert-generated message of the bank, $\chi^2(1, N=1{,}536{,}991)=1473.68$, $p<.001$, OR=1.29 (95%CI: 1.27-1.30). A total of 4,382 customers indicated they were not interested in the message (0.38%), much less compared to the bank's message, $\chi^2(1, N=1{,}536{,}991)=3933.89$, $p<.001$, OR=0.29 (95%CI: 0.28-0.30).

The message emphasizing forgetting read: "Forgot your subscriptions? All your subscriptions in one clear overview. Go to the overview!" This message was sent to 742,726 customers and generated 64,476 clicks (CTR = 8.68%), which is better than the expert-generated message of the bank, $\chi^2(1, N=1{,}122{,}215)=22.49$, $p<.001$, OR=1.03 (95%CI: 1.02-1.05). A total of 4,252 customers indicated they were not interested in the message (0.57%), much less compared to the bank's message, $\chi^2(1, N=1{,}122{,}215)=1586.95$, $p<.001$, OR=0.44 (95%CI: 0.42-0.46).

The message emphasizing possible savings read: "Reduce your spending? How much can you save by stopping unused subscriptions? See your subscriptions." This message was sent to 1,103,064 customers and generated 112,400 clicks (CTR = 10.19%), which is better than the expert-generated message of the bank, $\chi^2(1, N=1{,}482{,}553)=1011.15$, $p<.001$, OR=1.23 (95%CI: 1.22-1.25). A total of 6,373 customers indicated they were not interested in the message (0.58%), much less compared to the bank's message, $\chi^2(1, N=1{,}482{,}553)=1883.60$, $p<.001$, OR=0.45 (95%CI: 0.43-0.46).

The message emphasizing increased control over spending read: "More control over your money? All subscriptions in one overview, cancel what you do not use and save! See your subscriptions." This message was sent to 1,029,332 customers and generated 103,692 clicks (CTR = 10.07%), which is better than the expert-generated message of the bank, $\chi^2(1, N=1{,}408{,}821)=875.98$, $p<.001$, OR=1.22 (95%CI: 1.20-1.24). A total of 6,110 customers indicated they were not interested in the message (0.59%), much less compared to the bank's message, $\chi^2(1, N=1{,}408{,}821)=1720.62$, $p<.001$, OR=0.46 (95%CI: 0.44-0.48).

5. SI References

The Behavioural Insights Team, Target, Explore, Solution, Trial, Scale: An introduction to running simple behavioural insights projects. www.bi.team/publications/testsguide/ (2022).

R. B. Cialdini, The science of persuasion, *Sci. Am.* **284**, 76-81 (2001).

B. L. Duckworth , K. L. Milkman, A guide to megastudies. *P. Natl. Acad. Sci. U.S.A. Nexus*, **1**, pgac214 (2022).

S. J. Hoch, Who do we know: Predicting the interests and opinions of the American consumer." *J. Consum. Res.* **15**, 315-324 (1988).

C. K. Hsee, B. Ruan, The Pandora Effect: The power and peril of curiosity. *Psychol. Sci.* **27**, 659-666 (2016).

K. L. Milkman, et al., Megastudies improve the impact of applied behavioral science. *Nature* **600**, 478-483 (2021).

K. L. Milkman, et al., A 680,000-person megastudy of nudges to encourage vaccination in pharmacies. *P. Natl. Acad. Sci. U.S.A.* **119**, e2115126119 (2022).

R. E. Nisbett, T. D. Wilson. Telling more than we can know: Verbal reports on the mental processes. *Psychol. Rev.* **84**, 231-259 (1977).

B. Ruan, C. K. Hsee, Z. Y. Lu, The Teasing Effect: An underappreciated benefit of creating and resolving an uncertainty. *J. Mark. Res.*, **55**, 556-570 (2018).

R. H. Thaler, Psychology and savings policies. *Am. Econ. Rev.*, **84**, 186–192 (1994).

Z. Wojtowicz, G. Loewenstein, Curiosity and the economics of attention. *Curr. Opin. Behav. Sci.*, **35**, 135-140 (2020).
